# Adaptive Metafilm With Scalable Phase‐Tunable Emissivity for Energy‐Efficient Thermal Management

**DOI:** 10.1002/smll.74620

**Published:** 2026-07-25

**Authors:** James Laurence Ruello, Sudip Kumar Pal, Xiaoqing Yu, Joonyoung Yoo, Sihyun Jang, Gayeong Kim, Gunwoo Kim

**Affiliations:** ^1^ Department of Organic Materials and Textile Engineering Jeonbuk National University Jeonju‐si Jeonbuk Republic of Korea; ^2^ Department of Carbon Composites Convergence Materials Engineering Jeonbuk National University Jeonju‐si Jeonbuk Republic of Korea; ^3^ Department of Carbon Materials and Fiber Engineering Jeonbuk National University Jeonju‐si Jeonbuk Republic of Korea; ^4^ Department of JBNU‐KIST Industry Academia Convergence Research Jeonbuk National University Jeonju‐si Jeonbuk Republic of Korea

**Keywords:** adaptive metafilm (AMF), aluminum sputtering, multiple scattering, net‑zero thermal management

## Abstract

Efficient thermal management of enclosed systems through controlled manipulation of radiative cooling power represents a key functionality of adaptive composite metamaterials. Such materials enable precise regulation of heat emission under varying environmental conditions while maintaining scalability and durability. Here, we present an adaptive metafilm (AMF) that autonomously tunes its infrared (IR) emissivity near ambient temperature. The AMF is fabricated by embedding tungsten‐doped vanadium dioxide (W‐VO_2_) and indium tin oxide (ITO) nanoparticles in a low‐density polyethylene (LDPE) matrix, followed by bottom‑side aluminum sputtering to form a reflective back layer. Through systematic optimization of filler loading and film thickness, an optimal configuration (5 wt.% W‐VO_2_ | 1 wt.% ITO | 50 µm) was identified, providing maximal IR modulation by balancing absorption and multiple scattering. This design yields a transmittance tunability of up to 40.25% in the 9–11 µm wavelength range, enabling bidirectional switching between heat‐conserving and radiative‐cooling modes across ambient temperature transitions at 40°C. The flexible metafilm exhibits strong environmental stability, supported by a polyethylene‐based composite during repeated thermal cycling. Both optical and thermal evaluations demonstrate consistent thermoregulation without external power. This scalable, flexible AMF platform offers a practical route to net‐zero‐energy thermal regulation in buildings, enclosures, and mobile energy systems.

## Graphical abstract

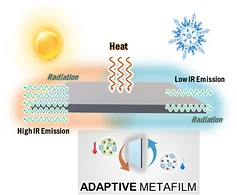

## 1 Introduction

The pervasive need for buildings and other indoor structures has become a ubiquitous and essential requirement in contemporary society. Conventional mechanical‐based cooling systems, such as air conditioners, present significant drawbacks: they are energy‐intensive, contribute to net heating, necessitate reliable electricity access, and frequently rely on refrigerants with detrimental environmental impacts, including ozone depletion and potent greenhouse gas emissions [1, 2, 3].

Radiative cooling is one of the emerging passive thermal management approaches that enables heat dissipation by emitting thermal radiation through the atmospheric transparency window (8–13 µm wavelength) directly into space [4, 5, 6]. This strategy has garnered tremendous interest as a sustainable alternative to conventional cooling technologies. Recent advances in composite materials [7, 8, 9, 10, 11, 12], porous structure [13, 14, 15], and multilayer photonic designs [16, 17, 18, 19], spectrally selective scattering have enabled passive radiative cooling systems coupled with efficiently reflecting incoming solar radiation. However, despite these developments, there remains a pressing need for cost‐effective, environmentally sound cooling solutions with adaptive cooling effects under varying climate conditions. During the colder season, the capability to transition a cooling surface into one that retains heat is crucial to avoid the energy penalty of subsequent heating. Addressing such dynamic thermal demands could significantly reduce energy consumption and mitigate greenhouse emissions, while also offering thermal stress relief in energy‐deprived scenarios.

Researchers have extensively investigated materials with temperature‐ or humidity‐triggered IR emissivity changes [17, 20, 21]. A prevalent approach employs a Fabry‐Perot cavity architecture, which is a resonant photonic structure that manipulates electromagnetic waves through multiple‐beam interference [22, 23, 24]. This design typically comprises an upper insulator‐to‐metal transition layer such as vanadium dioxides (VO_2_), an intermediate IR‐absorbing dielectric or polymeric layer that sets the prolonged optical path length, and a highly reflective bottom substrate such as an aluminum or indium tin oxide (ITO) layer that prevents IR transmission while reinforcing resonance [24, 25]. When the temperature exceeds the VO_2_ phase transition point, both the VO_2_ film and the reflective substrate exhibit metallic optical characteristics, and the structure behaves as a resonant cavity that confines photons within the IR‐absorbing spacer. In this combination, the cavity enhances photon confinement within the IR‐absorbing layer, thereby resulting in high‐yield phonon conversion for IR emission. As a result, the overall IR emissivity is substantially elevated, facilitating enhanced radiative thermal dissipation under thermally stressful environments. Despite these advances, achieving a metamaterial that combines scalability, near‐ambient phase transition temperatures, and highly tunable emissivity independent of incident light remains a significant challenge, particularly for large‐scale commercialization.

Composite VO_2_ particle‐polymer structures with a metallic backing constitute a scalable platform that delivers thermally adaptive IR emissivity [24, 26, 27, 28]. Above the phase‐transition temperature, VO_2_ particles (metallic phase) efficiently scatter infrared radiation, while the polymer matrix enhances IR absorption through a lengthened optical path from multiple scattering, yielding high effective emissivity. Because the particles are isotopically dispersed, the optical response is largely independent of incidence angle and polarization. Moreover, tungsten‐doped VO_2_ (W‐VO_2_) is readily produced as a powder, rather than as a thin film by the sputtering method, and exhibits a near‐ambient transition temperature, enabling practical room‐temperature applications. However, these composites require polymer matrices with tailored IR absorption, and achieving high‐solids, well‐dispersed VO_2_ loading remains challenging when relying solely on polymer compounding. Consequently, the approach is still underexplored, and near‐ambient phase‐transition operation suitable for room‐temperature use has yet to be demonstrated at scale.

W‐VO_2_ embedded in a polyethylene (PE) polymer matrix and co‐integrated with ITO nanoparticles represents a cutting‐edge approach to radiative cooling, leveraging synergistic material interactions and phonon‐mediated thermal dynamics. The engineered metafilm features an aluminum layer deposited by sputtering on one surface, which functions as a reflective backplane to suppress IR transmission and confine photons for high‐yield phonon conversion. The phase transition of W‐VO_2_ enables strong IR scattering and absorption within the surrounding PE matrix. The inclusion of ITO nanoparticles further broadens the emissivity tunability across the spectrum. The configuration of these design elements yields durable, high‐performing adaptive thermal regulation under varying environmental conditions, making it a promising candidate for next‐generation smart adaptive radiative cooling technologies.

Herein, a temperature‐adaptable metafilm for smart radiative cooling was engineered by embedding W‐VO_2_ and ITO nanoparticles within a PE matrix, followed by heat pressing to produce a thin composite film and subsequent aluminum sputtering on the bottom surface to serve as an effective IR‐reflective layer. The aluminum coating plays a dual role: (1) it converts transmittance of the composite polymer film into the reflection, thereby enabling IR optical tunability for thermoregulation, and (2) it transports high a flux of thermal energy along the surface owing to its high thermal conductivity. Under warm‐season conditions (*T* > *T*_p_ ≈ 40°C), W‐VO_2_ transitions to a metallic phase, leading to enhanced IR scattering and absorption within the polymer matrix. This results in a higher effective emissivity and promotes radiative heat dissipation through the 8–13 µm atmospheric window. On the contrary, during cold‐season conditions, W‐VO_2_ remains in its insulating phase, making the composite film relatively high IR‐transparent; this results in high IR‐reflective/low IR emissivity coupled with the reflective Al backing, which suppresses radiative losses, thereby retaining internal heat. This structure significantly amplifies IR emission within the atmospheric window, enabling up to 40% tunable emissivity for precise, temperature‐responsive radiative heat management. This high modulation depth allows year‐round thermal regulation with minimal energy input, offering a scalable pathway toward net‐zero‐energy thermal management in buildings, enclosures, and energy‑storage or vehicular systems.

## 2 Results and Discussion

### 2.1 Adaptive Radiative Cooling Concept and Fabrication

**FIGURE 1:**
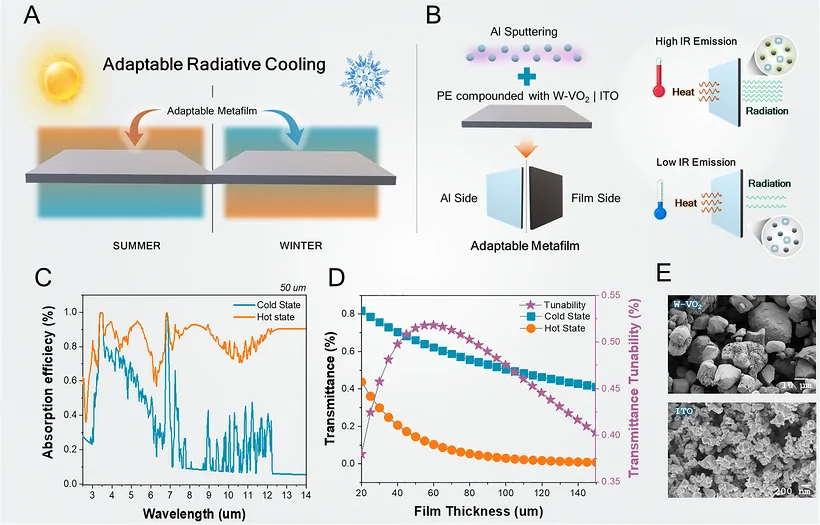
(a) Design and model of the adaptive metafilm that features adaptable system radiative cooling. (b) Schematic illustration of AMF of the reversible property of W‐VO_2_ (randomly embedded with ITO in the PE polymer matrix) from insulating to metallic with respect to temperature. (c) Calculated IR absorption spectra for the adaptive metafilm (50 µm thickness) across cold and hot states. (d) Simulated IR transmittance of the adaptive metafilm in the cold and hot states as a function of film thickness, along with the corresponding transmittance tunability. (e) SEM micrographs of W‐VO_2_ and ITO nanoparticles.

The thermoregulation mechanism responsible for maintaining constant core temperatures is critical in such engines and batteries (Figure 1a,b). An adaptive metafilm (AMF) composed of W‐VO_2_ and ITO dispersed within a PE matrix is systematically optimized in filler concentrations (particle scattering) and film thickness. Such optimization is critical for achieving reliable thermal management, particularly in systems that are susceptible to overheating during high‐temperature operation or excessive cooling in cold climates. Under hot conditions, materials engineered with high IR emissivity and low IR reflectivity facilitate efficient heat dissipation, thereby preventing thermal buildup in enclosed systems. Conversely, in cold environments, materials with low IR emissivity and high IR reflectivity help minimize radiative heat loss, preserving internal thermal stability. By dynamically balancing these emissive and reflective behaviors, the AMF activates adaptive heat exchange with the surroundings, ensuring consistent and energy‐efficient thermal regulation across diverse environmental conditions.

To realize this concept, we developed a simple yet effective fabrication strategy for metamaterials capable of adaptive radiative cooling. The proposed metafilm autonomously adjusts its IR emissivity in response to temperature fluctuations, inducing dynamic thermoregulation under varying environmental conditions. As illustrated in Figure S1, the AMF is produced by compounding an optimized ratio of W‐VO_2_ and ITO within a low‐density polyethylene (LDPE) matrix, followed by aluminum sputter deposition to form a reflective back layer. The aluminum coating process requires careful control because prolonged exposure to heat during the sputtering process can thermally degrade the polymer matrix, while insufficient deposition time may result in incomplete surface coverage and reduced IR reflectivity. When properly optimized, the aluminum layer converts the intrinsic IR transmittance tunability of the composite into tunable IR reflectance, establishing a balance between emissive and reflective modulation, characteristic of a pseudo‐Fabry‐Pérot cavity formed by the particle‐layer architecture.

Among the active constituents, W‐VO_2_ serves as a thermochromic component that undergoes a reversible insulator‐to‐metal transition (IMT) near ambient temperature, dramatically altering its optical properties [29, 30]. In its insulating monoclinic phase, W‐VO_2_ exhibits low IR scattering, enabling efficient transmittance of thermal infrared radiation through the composite when coupled with a low‐IR‐absorption polymer matrix such as PE [27]. Above its transition temperature, the metallic rutile phase of W‐VO_2_ strongly scatters IR radiation, increasing IR absorption in the polymer matrix, which results in high emissivity of the composite. Tungsten‐doped alloy structure shifts the IMT temperature of VO_2_ from ∼68°C to a lower temperature (1.4 wt. % tungsten for around 40°C IMT temperature), aligning the switching thermal behavior with practical environmental conditions. The composite design further leverages ITO nanoparticles to enhance optical modulation. The aluminum base layer enhances photon confinement, converting IR transmittance of the composite into IR reflectance below the transition temperature, while maintaining high IR emissivity at temperatures above the IMT.

The IR transmittance of AMF structures can be described, to first order, by a Beer–Lambert model that accounts for front‐surface reflection and absorption within the composite medium,

(1) $$\tau =\left(1-\rho \right)e^{-\alpha L}$$

where ρ is the IR reflectance of the AMF surface, α is the effective absorption coefficient of the composite (polymer matrix plus inclusions), *L* is the optical path length of IR in the polymer matrix, and τ is the IR transmittance. Equation (1) highlights the exponential suppression of transmitted IR radiation with increasing optical path length, implying that thickness control is a primary lever for maximizing temperature‐dependent radiative switching in thermally AMF.

To explicitly quantify switching performance, we define the transmittance tunability (Δτ) between the low‐temperature (LT) and high‐temperature (HT) states as,

(2) $$\left(1-\rho \right)\;\left(e^{-\alpha \beta_{1}L}-e^{-\alpha \beta_{2}L}\right)=\Delta \tau_{AMFw/oAl}=\Delta \rho$$

and, after aluminum deposition (AMF w/ Al), as

(3) $$\left(1-\rho \right)\;\left(e^{-\alpha \beta_{3}L}-e^{-\alpha \beta_{4}L}\right)=\Delta \rho_{AMFw/Al}=\Delta \varepsilon$$

where β_1_ and β_2_ represent the effective optical‐path factors for at low and high temperatures before aluminum sputtering, while β_3_ and β_4_ are the corresponding factors after sputtering because these parameters capture changes in effective path length and optical feedback associated with the metal‐backed configuration. Here *L* is the physical film thickness. Δτ_*AMF* *w*/*o* *Al*_ represents the IR transmittance tunability of the AMF between the low‐ and high‐temperature states. After depositing an optically thick aluminum layer that functions as an ∼100% IR reflector, the film becomes effectively opaque in the IR (*τ* ≈ 0). Under this condition, the switching that was previously expressed as transmittance modulation is manifested primarily as reflectance modulation. Thus Δτ_*AMF* *w*/*o* *Al*_ corresponds to the reflectance tunability Δρ. Accordingly, Δρ_*AMF* *w*/ *Al*_ denotes the reflectance change after aluminum deposition. Moreover, because the Al‐backed film exhibits negligible transmittance, the emissivity change between thermal states can be obtained directly from reflectance via Kirchhoff's law (for an opaque film, *ε* ≈ 1‐*ρ*). The transmission is negligible (*τ* ≈ 0) due to the optically thick aluminum layer. Under this opaque condition, Kirchhoff's law permits the simplified relation *ε* ≈ 1 − *ρ*. We note that in systems with partial transmission or significant scattering, the full *ε* = 1 − *ρ* − *τ* formulation would be necessary, but in our case *τ* ≈ 0, justifying the approximation. Therefore, Δρ_*AMF* *w*/ *Al*_ corresponds to Δε, which quantifies the emissivity tunability between low and high temperatures. This formulation enables a direct evaluation of how intrinsic material properties and film thickness govern the overall spectral switching performance.

To connect this analytical framework to the experimentally relevant thickness window, the absorption efficiency was calculated theoretically for multiple film thicknesses using an effective medium theory (EMT) implemented in MATLAB. Using the temperature‐dependent optical constants of the constituent phases, the effective complex refractive index of the composite has been calculated in the LT and HT states and then evaluated the corresponding absorption response as a function of thickness. This analysis shows that a film thickness of L≈50 µm yields the largest LT‐HT absorption efficiency tunability (Figure 1c) compared with thinner or thicker films (20, 80, 100, 120, and 150 µm (Figure S2). Building on these EMT‐derived optical constants, theoretically IR transmittance at 10 µm has been calculated for each thickness in both thermal states and extracted the thickness‐dependent transmittance tunability, Δτ (Figure 1d). The results identify an optimum thickness window of approximately 50–70 µm, where the transmittance modulation reaches its maximum. In this regime, increasing *L* amplifies phase‐dependent attenuation differences because W‑VO_2_ is insulating (monoclinic) below the transition temperature, permitting moderate IR transmission, whereas above the transition, it becomes metallic (rutile), increasing free‐carrier absorption and modifying the complex refractive index to suppress τ. However, for *L >* 60 µm, both LT and HT states approach the optically thick limit over overlapping IR bands, so transmission is strongly suppressed in both cases, causing the differential transmission collapses, reducing Δ*τ*. In practical terms, overly thick films absorb too strongly regardless of phase, which diminishes the modulation contrast.

Following aluminum sputtering, the AMF switches from a transmission‐based mechanism to reflection‐based thermal control. The aluminum layer acts as a broadband IR mirror, suppressing transmission and enhancing outward IR reflectivity depending on the phase of W‑VO_2_. Yet the core mechanism remains thickness‐dependent: infrared photons still interact volumetrically with the W‑VO_2_ layer before being reflected, and the phase‐dependent emissivity contrast determines the thermal switching. Figure 1e presents the SEM images of W‐VO_2_ and ITO nanoparticles, revealing their nanoscale morphology that is essential for consistent and efficient IR interaction. As validated by simulations in Figures S2 and S3, thin films offer insufficient interaction volume for strong modulation, while overly thick films show high infrared absorption in both thermal states, diminishing the net transmittance difference.

### 2.2 Physical and Chemical Characterization

**FIGURE 2:**
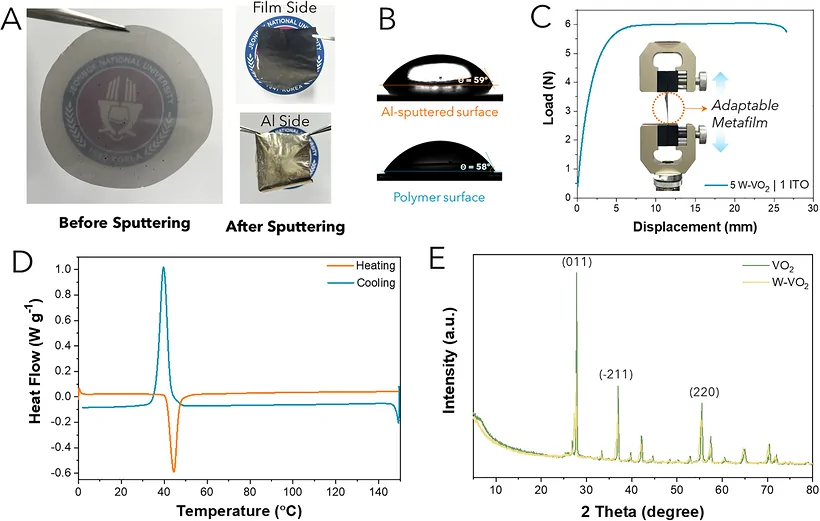
(a) Photograph illustration of the AMF film before and after Al sputtering (film and metal side). (b) Contact angle analysis of AMF material's Al‐sputtered and polymer surfaces. (c) Mechanical tensile properties of AMF material with corresponding tensile test image. (d) DSC and (e) XRD analysis of W‐VO_2_ used in the AMF material.

Figure 2a illustrates the AMF prior to and following aluminum sputtering, presenting the polymer‐exposed side (top right) alongside the reflective aluminum‐coated side (bottom right). The left image highlights the semi‐transparency of the pristine film, allowing the logo beneath to be visible through the composite matrix. This visual transformation confirms successful deposition of the aluminum layer, which imparts reflectance‐based modulation capability to the film. The contrasting optical appearances between the two sides demonstrate the dual‐mode character of the AMF: (1) optically transparent and emissive on one side, and (2) thermally reflective on the other. Figure S4a,b shows SEM micrographs of the aluminum‐sputtered surface and cross‐section of the AMF material. The aluminum layer appears uniformly flat and free from surface irregularities or morphological defects, confirming that the sputtering process preserves the integrity of the polymer substrate. The cross‐sectional image reveals a thin, conformal aluminum coating on the LDPE matrix, indicating that deposition occurred without inducing significant film thickening or surface distortion. These phenomena demonstrate that the controlled aluminum sputtering process produces a continuous and reflective metallic layer essential for stable optical and thermal performance in adaptive radiative cooling.

Water contact angle analysis evaluates surface wettability, offering insight into the material's interfacial energy. The wettability of AMF was quantified through water contact‐angle measurements (Figure 2b). The Al‐sputtered and non‐sputtered polymer surfaces exhibit nearly identical contact angles of 59 and 58°, respectively. The modest wettability of the Al‐coated surface is attributed to the spontaneous formation of a thin Al_2_O_3_ layer upon exposure to air. In contrast, pristine LDPE is typically hydrophobic, displaying contact angles near 90°. The observed decrease in contact angle for the polymer surface is likely due to the sputtering process that can oxidize the LDPE layer.

High flexibility and mechanical durability are essential for a metafilm to fully exploit its potential and ensure long‐term reliability. The tensile behavior of the AMF is presented in Figure 2c, where load (N) is plotted as a function of displacement (mm). The initial linear region of the stress‐strain curve reflects elastic deformation, where the slope defines the Young's modulus and indicates the intrinsic stiffness of the film. Once the yield point is exceeded (around 6 N), the material enters a plastic deformation regime marked by substantial elongation between 5 and 25 mm under a relatively stable load. This behavior signifies excellent ductility, which allows the film to deform permanently without immediate failure. Beyond this range, a gradual decline in load suggests the initiation of necking and localized structural weakening that eventually leads to fracture. The composite containing 5 wt.% W‐VO_2_ and 1 wt.% ITO demonstrates a favorable mechanical profile, combining high stiffness with remarkable stretchability, even after the sputtering process. This balance is essential for maintaining performance stability in applications that require frequent mechanical flexing. The ability to withstand bending, stretching, or rolling without significant mechanical degradation supports the long‐term reliability of the metafilm in adaptive radiative cooling systems.

Thermal and structural analyses are important for verifying the temperature‐driven phase transition and crystalline structure of W‐VO_2_, as these properties directly determine its optical and emissive switching behavior. The DSC curve (Figure 2d) shows a distinct endothermic peak around 44°C on heating and an exothermic peak near 40°C on cooling, confirming a reversible insulator‑to‑metal transition. The lowered transition temperature and narrow hysteresis of approximately 4°C demonstrate effective tungsten doping and fast, efficient switching, making the material well‑suited for near‑ambient thermochromic applications. On the other hand, the XRD patterns of pristine VO_2_ and W‑VO_2_ (Figure 2e) confirm that both materials crystallize in the pure monoclinic M1 phase, with characteristic diffraction peaks at (011), (200), and (220) matching JCPDS card no. 72–0514 [31, 32], and no secondary phases were detected. While pristine VO_2_ shows sharp, high‐intensity reflections indicative of excellent crystallinity, the W‑VO_2_ pattern exhibits noticeably reduced peak intensities and broader peaks, particularly the dominant (011) reflection, consistent with successful substitutional doping that introduces lattice microstrain and distortion without compromising overall phase purity. This structural perturbation arises from W^6+^ replacing V^4+^, which generates excess electrons that weaken V‐V dimerization along the monoclinic chains and lower the energetic barrier for the metal‐insulator transition, enabling thermochromic switching closer to ambient conditions for adaptive infrared applications [32, 33].

### 2.3 Thermo‐Responsive Optical Properties

**FIGURE 3:**
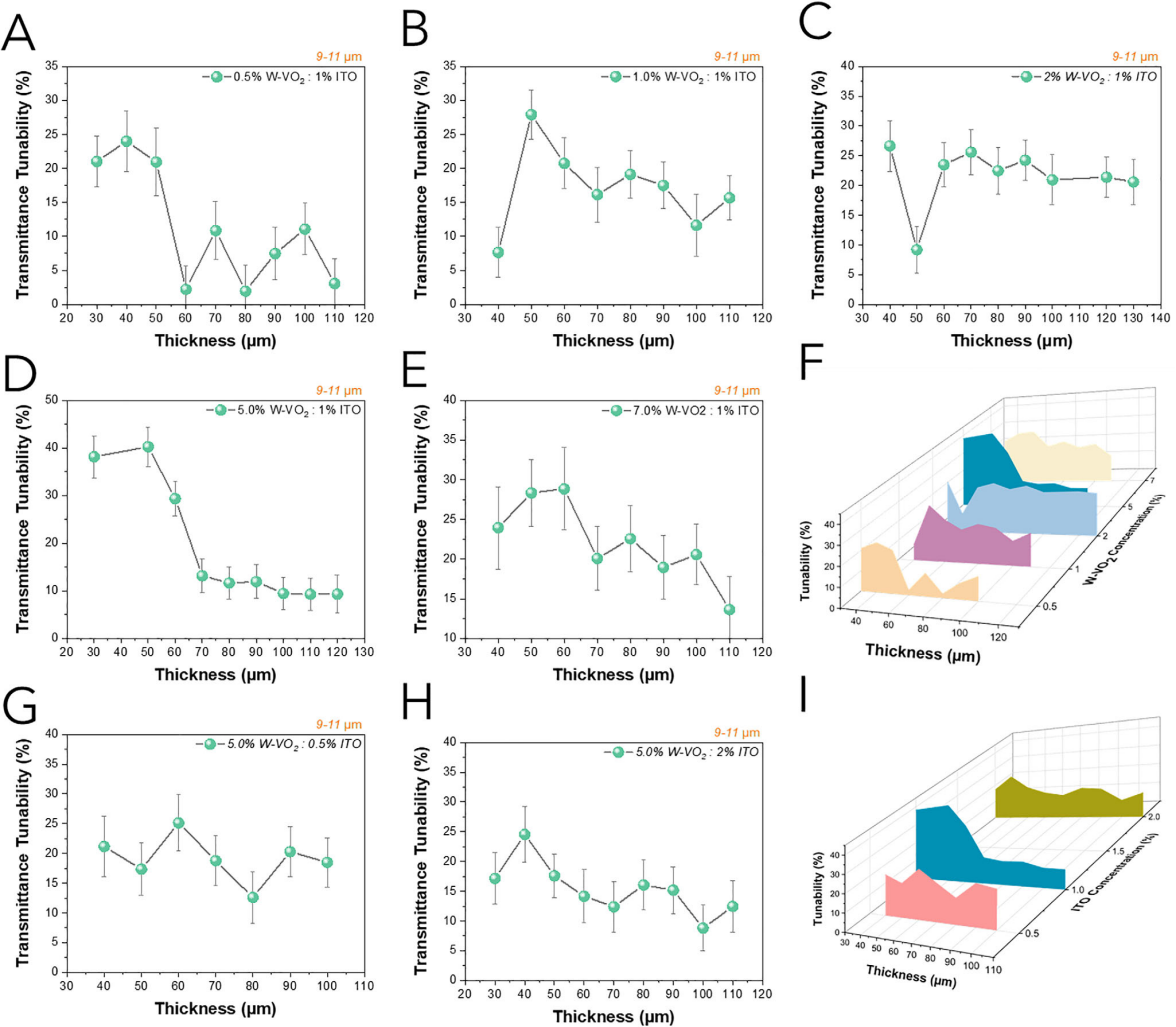
Thickness‐ and composition‐dependent transmittance tunability of composite films in the 9–11 µm region. (a–e) Tunability as a function of film thickness for different W‐VO_2_ concentrations. (f) 3D mapping of transmittance tunability versus film thickness and W‐VO_2_ concentration derived from (a–e). (g–h) Tunability as a function of film thickness for varying ITO concentrations. (i) 3D mapping of tunability versus film thickness and ITO concentration corresponding to (g–h).

The IR transmittance tunability of the AMF is governed by filler concentration and the film thickness, which together determine the effective optical interaction volume and the phase‐dependent absorption contrast of the composite. To elucidate these effects, IR transmittance tunability of the AMF without an aluminum layer was systematically investigated as a function of W‐VO_2_ loading, ITO content, and film thickness. Figures S5–S12 present the temperature‐dependent transmittance spectra under hot and cold states, while Figure 3a–e summarizes the corresponding tunability in the IR atmospheric window at a fixed ITO concentration of 1 wt.% and varying W‐VO_2_ content. Across all compositions, the modulation exhibits a non‐monotonic dependence on thickness, indicating that IR tunability is not governed by thickness alone but instead emerges from an optimized balance between absorption strength and phase sensitivity.

The concentration of W‐VO_2_ plays a crucial role in determining the optical tunability of the film. Although all samples were heated sufficiently beyond the IMT threshold to ensure complete phase transition, low concentrations of W‐VO_2_ nanoparticles lead to limited scattering and absorption contrast across thermal states. This is because the sparsely distributed nanoparticles within the polymer matrix provide insufficient optical interaction volume in the 9–11 µm range, reducing the ability of the film to modulate transmittance effectively. In essence, fewer particles result in less differential scattering between both phases, thereby lowering the overall modulation depth. Moreover, according to the reformulated Beer‐Lambert model, when the absorption contrast between β_1_ and β_2_ is small, both exponential terms decay rapidly with increasing *L*, resulting in similar attenuation in both phases. This leads to a significant drop in overall transmittance but without meaningful tunability, which is an indication that longer optical paths, when not paired with sufficient phase‐switching material, ultimately reduce dynamic modulation efficiency. As the W‐VO_2_ concentration increases to 5 wt.%, the composite enters an optimal modulation regime. At this loading, the nanoparticle density becomes sufficient to establish a pronounced absorption contrast between the insulating and metallic phases, leading to distinctly different effective absorption coefficients (β_1_ and β_2_). This contrast amplifies the difference in exponential attenuation between thermal states, maximizing the transmittance modulation for a given optical path length. Importantly, this enhancement does not arise from excessive absorption but from an efficient redistribution of absorption strength across phases. The AMF with 5 wt.% W‐VO_2_ and a thickness of 50 µm (Figure 3d) achieves a maximum tunability (Δτ ≈  40.25%), which closely matches numerical simulations (Figure 1d) predicting an optimal thickness range of 50–60 µm. This agreement confirms that the experimentally observed optimum reflects an intrinsic balance between absorption contrast and optical path length rather than a processing artifact.

Beyond this optimum concentration, further increasing the W‐VO_2_ content to 7 wt.% results in a deterioration of tunability. At high loadings, the composite approaches an absorption‐saturated regime in which both the insulating and metallic states exhibit strong attenuation in 9–11 µm wavelength. Under these conditions, the absorption coefficients β_1_ and β_2_ become large and increasingly similar, causing the transmittance levels of the two phases to converge. As a result, the differential modulation collapses despite the presence of a larger amount of thermochromic material. In addition, excessive nanoparticle loading can promote agglomeration, enhancing multiple scattering and internal IR trapping within the polymer matrix. These effects further suppress transmittance in both thermal states and reduce the effective phase contrast, particularly at larger thickness.

The combined influence of W‐VO_2_ concentration and film thickness is captured in the 3D surface plot shown in Figure 3f, which maps the transmittance tunability across a wide compositional and structural parameter space. A well‐defined maximum emerges at approximately 5 wt.% W‐VO_2_ and a thickness of 50–60 µm, confirming the existence of a narrow design window in which strong phase‐dependent absorption contrast and sufficient optical path length are simultaneously achieved. At lower concentrations, tunability remains uniformly low regardless of thickness due to insufficient absorption contrast, while at higher concentrations the system transitions into an absorption‐dominated regime where phase sensitivity is effectively lost. The non‐monotonic thickness dependence observed across most concentrations further highlights that increasing thickness initially enhances light‐matter interaction but ultimately leads to comparable attenuation in both phases once absorption saturation is reached.

Importantly, these optical and thermal measurements confirm that the adaptive metafilm exhibits bidirectional functionality at the material level. In the insulating phase (<40°C), the composite film coupled with the aluminum backing suppresses radiative losses, thereby retaining heat. In the metallic phase (>40°C), enhanced IR scattering and absorption promote radiative cooling through the atmospheric window. Thus, while full system‑level demonstrations are beyond the scope of this study, the experimentally observed emissivity modulation directly establishes the dual‑mode capability of the metafilm and its potential for bidirectional thermal regulation.

The role of ITO in the composite film can be understood from an optical path length perspective within the reformulated Beer‐Lambert framework, analogous to the behavior observed for varying W‐VO_2_ concentration. At low ITO loading (0.5 wt.%; Figure 3g), the sparse distribution of ITO particles leads to weak and spatially non‐uniform infrared absorption, resulting in a short and locally varying effective optical path length. This optical heterogeneity causes the phase‐dependent attenuation terms to evolve inconsistently with thickness, giving rise to unstable and fluctuating transmittance modulation. At an intermediate ITO concentration of 1 wt. %, the optical path length is more uniformly defined throughout the composite due to the continuous presence of optically active ITO domains. In this balanced regime, ITO provides moderate broadband infrared attenuation while remaining optically complementary to W‐VO_2_. Moreover, the presence of ITO is expected to amplify the effective infrared scattering associated with W‐VO_2_ by increasing refractive‐index contrast and extending the optical path length through scattering‐assisted attenuation. This coupled optical interaction enhances the phase‐dependent contrast between the insulating and metallic states without saturating absorption, resulting in smooth, stable, and thickness‐tolerant transmittance tunability that closely mirrors the optimal W‐VO_2_ concentration regime. When the ITO concentration is further increased to 2 wt.% (Figure 3h), the system transitions into an absorption‐dominated regime. Strong free‐carrier absorption and enhanced multiple scattering led to an excessively large effective optical path length, causing both thermal states to undergo similarly strong exponential decay. As a result, the transmittance contrast between phases diminishes rapidly with increasing thickness, producing a monotonic decline in tunability. The thickness‐dependent trends summarized in the 3D mapping (Figure 3i) confirm that ITO loading critically controls both the magnitude and uniformity of the effective optical path length. Maximal IR transmittance modulation is achieved only within a narrow ITO concentration window, where absorption is sufficient to enhance phase contrast without driving the composite into an absorption‐saturated regime.

**FIGURE 4:**
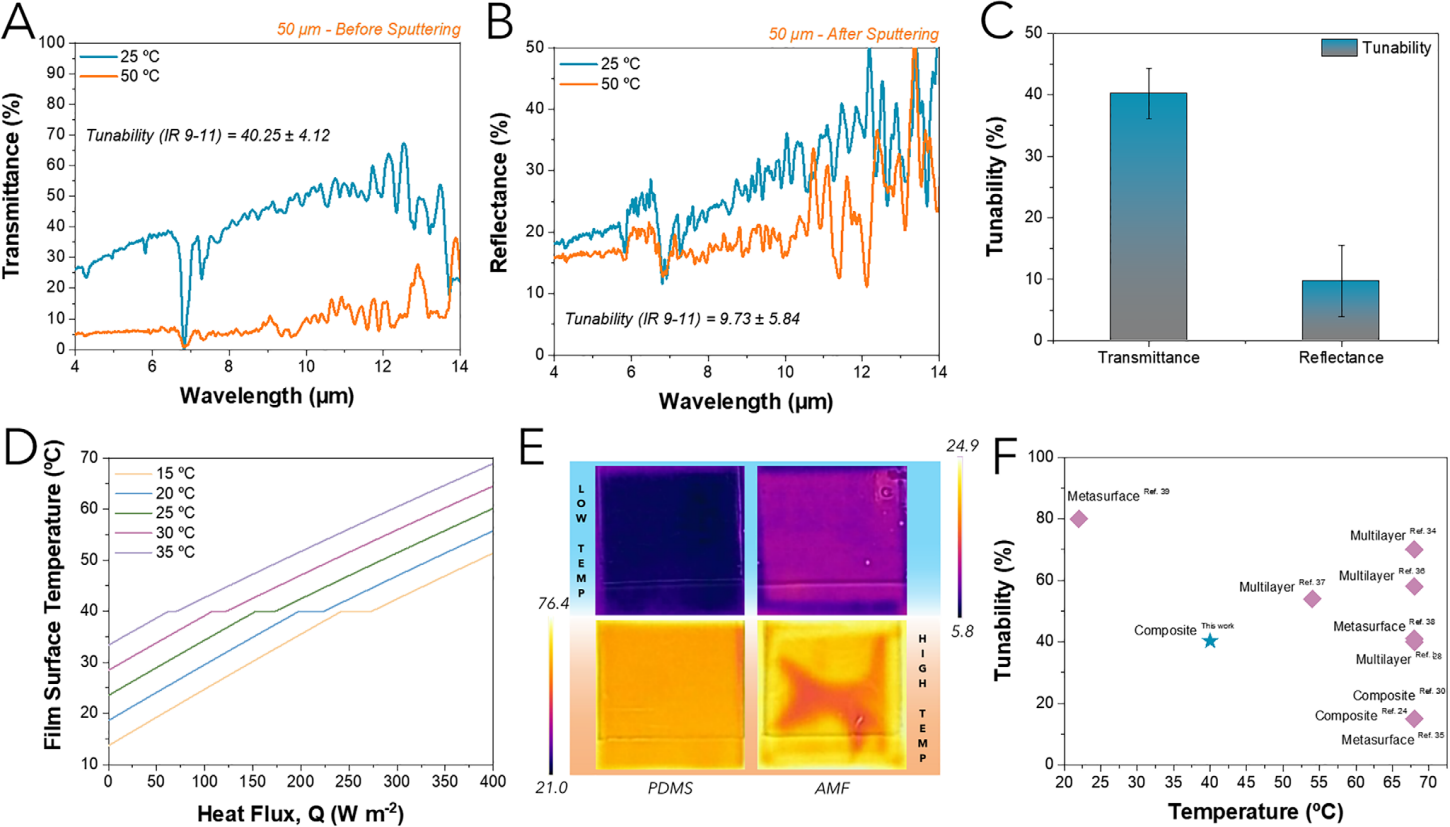
(a) IR spectral transmittance of optimized AMF before Al sputtering at room and high temperatures. (b) IR spectral reflectance of optimized AMF after Al sputtering at room and high temperatures. (c) Tunability histogram of AMF material. (d) Calculated film surface temperature (10°C–35°C) versus heat flux for AMF under varying heat flux conditions. (e) Thermal images of PDMS and AMF on metal samples at high and low temperature exposures. (f) Comparison of tunability versus transition temperature for the adaptive metafilm developed in this work and previously reported VO_2_‑based emitters [24, 28, 30, 34, 35, 36, 37, 38, 39].

Evaluation of IR control functionality is presented in Figure 4a–c, which shows the transmittance and reflectance behavior of the optimized AMF formulation (5 wt.% W‐VO_2_ and 1 wt.% ITO at a thickness of 50 µm) before and after Al sputtering.

Figure 4a presents the temperature‐dependent IR transmittance spectra of the adaptive metafilm prior to aluminum sputtering. A pronounced thermochromic modulation is observed across the 9–11 µm atmospheric window, where the film exhibits high transmittance at low temperature and significantly reduced transmittance at elevated temperature. This behavior originates from the transition temperature of the W‐VO_2_ nanoparticles. In the insulating state, W‐VO_2_ interacts weakly with IR radiation, allowing infrared photons to transmit through the film. Upon heating above the transition temperature, W‐VO_2_ transitions into its metallic phase, where enhanced free‐carrier absorption and reflection strongly attenuate IR transmission. Following aluminum sputtering, the optical modulation mechanism transitions from transmission‐dominated to reflection‐dominated control, as shown in Figure 4b. This behavior can be quantitatively described using the modified Beer–Lambert formulation for the sputtered AMF (Equation 3). In this configuration, infrared radiation traverses the composite film twice (once upon incidence and once after reflection from the aluminum backing), effectively doubling the optical path length to *2L*. As a result, a sputtered AMF with a physical thickness of ∼50 µm experiences an optical interaction equivalent to a ∼100 µm free‐standing film without aluminum. This double‐pass light‐matter interaction establishes a pseudo‐Fabry‐Pérot optical configuration, in which the aluminum layer serves as a reflective boundary while the composite metafilm functions as a phase‐switchable infrared transmittance medium. Unlike conventional Fabry–Pérot cavities that rely on coherent interference and resonance effects, the present structure operates in a non‐resonant, path‐length‐dominated regime, where infrared modulation is governed by reflection‐assisted extension of the optical path length rather than cavity resonance. The reflected infrared radiation re‐enters the composite layer, effectively doubling the optical path length, which enhances the phase‐dependent transmittance contrast by amplifying the exponential attenuation difference between the insulating and metallic states of W‐VO_2_. As a result, emissivity modulation is strengthened without relying on interference effects.

Consequently, the measured reflectance tunability after sputtering (∼12%–15%, Figure 4c) closely parallels the transmittance tunability observed for the 100 µm‐thick composite film prior to sputtering (Figure 3d). Although the absolute modulation amplitude is reduced compared to transmission‐mode operation due to the intrinsically high reflectance baseline of aluminum, the phase‐dependent exponential attenuation remains preserved. At elevated temperatures, the metallic phase of W‐VO_2_ strongly interacts with infrared radiation, and in conjunction with the reflective aluminum layer, promotes enhanced internal absorption and efficient outward radiative emission. Conversely, at low temperatures, reduced IR interaction in the insulating state, together with the aluminum reflector, suppresses emission and favors infrared retention. The emissivity origin of this behavior is further confirmed in Figure S13, where a pristine PE film with aluminum sputtering shows negligible reflectance modulation in the 9–11 µm range. This comparison confirms that the observed tunability arises from the thermo‐responsive composite rather than from the polymer matrix or aluminum coating alone. The consistency between post‐sputtering reflectance tunability and pre‐sputtering transmittance trends validates the Beer‐Lambert‐governed optical‐path‐length argument and demonstrates that the sputtered AMF operates as a thermally adaptive surface capable of autonomously switching between radiative cooling under hot conditions and heat conservation under cold conditions.

Moreover, the mechanical durability of the AMF under cyclic deformation was evaluated through repeated bending tests by manually folding and unfolding the film over 100 cycles. As shown in Figure S14a, the IR reflectance tunability remained virtually unchanged, confirming that the emissivity modulation performance of the AMF is mechanically robust and stable under repeated flexing. This durability is essential for potential real‐world applications for adaptable thermal management, where frequent mechanical stress is expected.

The UV aging stability (Figure S14b) of the adaptive metafilm (AMF) was evaluated by comparing its mid‐infrared emissivity before and after prolonged 48 h UV (∼10 W/m^2^) exposure, as shown in Figure S13b. The emissivity in the 9–11 µm range exhibits only a slight increase after aging, from ∼70% to ∼72% at ∼20°C and from ∼78% to ∼80% at ∼50°C. Importantly, the temperature‐dependent emissivity contrast is largely preserved, indicating that the thermochromic response of W‐VO_2_ remains stable.

### 2.4 Thermal Application

Theoretical calculations were performed to demonstrate, quantify, and validate how emissivity switching in W‐VO_2_ enables temperature‐dependent thermal regulation, a key mechanism underlying the energy‐saving functionality of the AMF. These calculations are based on the following heat balance equations:

(4) $$Q=\sigma \times \left(T_{1}^{4}-T_{2}^{4}\right)\times \varepsilon \left(T_{1}\right)+C_{d}\times \left(T_{1}-T_{2}\right)$$

(5) $$\begin{array}{cc} & \sigma \times \left(T_{1}^{4}-T_{2}^{4}\right)\times \varepsilon \left(T_{1}\right)+C_{d}\times \left(T_{1}-T_{2}\right)\\ & =C_{v}\times \left(T_{2}-T_{a}\right)+0.9\times \sigma \times \left(T_{2}^{4}-T_{a}^{4}\right)+0.1\times \sigma \times \left(T_{2}^{4}-4K^{4}\right) \end{array}$$

where *Q* is given heat flux, *C_d_* and *C_v_* is the conduction and convection co‐efficient, *T*_1_ is the thin film temperature, *T*_2_ is the surface temperature of the set‐up, *T_a_* is the ambient temperature, σ is the Stefan–Boltzmann constant, and ε is the emissivity of the materials.

Figure 4d presents the simulated surface temperature of the window metafilm as a function of applied heat flux under different ambient temperatures (*T*_a_ = 15°C–35°C). The model incorporates coupled heat transfer (conduction, convection, and thermal radiation) together with a temperature‐dependent emissivity that captures the thermochromic response of W‐VO_2_. Specifically, the emissivity is set to *ε* = 0.1 below 40°C and *ε* = 0.9 above this threshold, corresponding to the insulating‐to‐metallic phase transition of W‐VO_2_. A distinct change in the slope of each temperature‐heat‐flux curve is observed as the surface temperature approaches ∼40°C, marking the onset of emissivity switching. Below this transition temperature, the low‐emissivity state suppresses radiative heat loss, causing the surface temperature to rise rapidly with increasing heat flux. Once the transition is reached, the abrupt increase in emissivity substantially enhances radiative cooling, thereby moderating further temperature rise despite continued thermal input. At an ambient temperature of 25°C, this transition occurs at an applied heat flux of approximately 150 W m^−2^, which is consistent with the experimentally observed infrared response of the metafilm. Notably, this same heat flux was employed during IR measurements to drive the AMF into the high‐temperature state, ensuring that W‐VO_2_ fully transitioned into its metallic phase prior to optical characterization. The close agreement between simulation and experiment confirms that the chosen heating condition reliably activates the thermochromic transition and validates the use of the thermal model to interpret the measured IR emissivity and transmittance behavior.

The thermal images in Figure 4e and Figure S15 directly visualize the temperature‐responsive emissivity modulation of the AMF in comparison with PDMS under low‐ and high‐temperature conditions. In the low‐temperature state (top row), PDMS appears dark purple, consistent with its intrinsically high emissivity (*ε* ≈ 0.9), which promotes efficient radiative heat loss and a cooler apparent surface. Notably, the AMF exhibits a magenta tone whose temperature reading is far from PDMS, indicating reduced infrared emission at low temperature. This behavior reflects the insulating monoclinic phase of W‐VO_2_, which weakly interacts with IR radiation and favors heat retention.

At elevated temperatures (bottom row), the infrared contrast reverses, wherein PDMS remains bright due to its persistently high emissivity. In contrast, the AMF now appears bright yellow‐orange (temperature closer to PDMS), signaling a substantial increase in emissivity. This transition arises from the phase change of W‐VO_2_ into its rutile phase, which modifies its optical constants and enhances IR absorption and scattering. As a result, the AMF efficiently dissipates heat through radiative emission at high temperature while suppressing emission at low temperature, confirming its thermochromic switching functionality. This adaptive emissivity behavior is consistent with the Beer‐Lambert‐based optical framework introduced earlier. At low temperature, weak IR interaction in the insulating state leads to limited attenuation and suppressed emission, whereas at high temperature, the metallic state enhances phase‐dependent attenuation and, in the aluminum‐backed configuration, benefits from an effectively extended optical path length. The resulting difference in exponential attenuation between thermal states underpins the observed tunability. Importantly, the AMF maintains a surface temperature close to ambient in both regimes, in contrast to PDMS, demonstrating its ability to conserve heat under cold conditions and release heat under hot conditions. While net cooling power or absolute temperature reduction was not measured in this study, the emissivity modulation and IR thermography provide direct evidence of radiative cooling behavior at the material level. Future work will extend to quantitative system‑level cooling power measurements in applications such as batteries and mobility systems.

Furthermore, the spatially uniform thermal profiles of the AMF indicate a consistent phase transition across the surface, likely facilitated by the presence of ITO nanoparticles that promote homogeneous optical and thermal interactions. The close agreement between these experimental thermal images, the numerical simulations, and the optical transmittance measurements reinforces the robustness of the proposed theoretical model. From an application perspective, this self‐regulating emissivity response suggests strong potential for passive thermal shielding in automotive environments, particularly for components such as battery enclosures, electronic modules, or vehicle surfaces exposed to ambient heat fluctuations. With appropriate encapsulation or integration onto thermally robust substrates, the AMF could mitigate external heat loading without additional energy input, offering a promising route toward energy‐efficient thermal management in next‐generation vehicles. Beyond these application‑oriented advantages, comparative analysis with previously reported VO_2_‑based emitters (Figure 4f) further underscores the uniqueness of this design: unlike multilayer or membrane structures that require complex fabrication and higher transition temperatures, the single‑layer AMF with aluminum sputtering achieves high tunability (∼40%) at ∼40°C. This combination of fabrication simplicity, thin architecture, and effective performance at moderate temperatures highlights its practicality and scalability for real‑world thermal regulation.

## 3 Conclusion

In summary, a scalable adaptive metafilm (AMF) was developed by embedding W‐doped VO_2_ and ITO nanoparticles into a PE matrix and integrating it with a metallic reflector. This multilayer architecture enables reversible modulation of IR transmittance and reflectance, governed by the thermally induced insulator‐to‐metal phase transition of W‐VO_2_. Systematic tuning of thickness and filler content revealed optimized configurations that balance emissivity contrast, switching uniformity, and mechanical flexibility. The AMF exhibits a high transmittance tunability of ∼40%, thermally adaptive emissivity switching, and stable performance even after repeated mechanical deformation. Through combined experimental validation and numerical simulation, the film demonstrates programmable IR behavior, transiting between low‐emissivity (heat‐conserving) and high‐emissivity (cooling) states, making it uniquely suited for bidirectional passive thermal regulation. Unlike conventional materials with fixed emissivity, the AMF autonomously adjusts its radiative output in response to ambient temperature. This work offers a new class of scalable, flexible, and energy‐efficient surfaces for next‐generation smart thermal metamaterials, adaptive building envelopes, cooling systems, and sustainable IR‐regulating technologies. The integration of tunable optics with robust mechanical and thermal resilience highlights a promising route toward intelligent materials engineered for dynamic environmental interactions.

## 4 Materials and Methods

### 4.1 Materials

LDPE was purchased from Hanhwa Chemicals. Tungsten‐doped vanadium dioxide (W‐VO_2_) was procured from Guangzhou Hongwu Material Technology Co., Ltd., while ITO was obtained from Dongguan SAT Nano Technology Material Co.

### 4.2 Preparation of ITO/W‐VO_2_‐Incorporated Film

ITO and W‐VO_2_ nanoparticles were first individually ball milled (Samheung Energy, Model SH‐Ball‐300‐1) to eliminate agglomerates and achieve a consistent particle size distribution. The nanoparticles were then vacuum dried (Samheung Energy, Model SH‐VDO‐08NG) to remove moisture content. Composite formulations were prepared by blending the dried nanoparticles with LDPE at defined ratios: specifically, 1 wt% ITO with varying W‐VO_2_ concentrations (0.5, 1, 2, 5, and 7 wt%), and alternatively 5 wt% W‐VO_2_ with varying ITO concentrations (0.5, 1, and 2 wt%). These mixtures were processed through multiple passes in a single‐screw extruder (SJ15Lab) to ensure uniform dispersion and integration within the LDPE matrix. Thin films were subsequently fabricated via heat pressing (Heating Plate Tester, Model QM900M), yielding homogeneously dispersed polymer‐inorganic composites optimized for adaptive radiative cooling applications.

### 4.3 Preparation of the Adaptive Metafilm

The fabricated thin films were coated with a uniform aluminum layer by sputtering (JVAC, SPM‐1). This process forms a conformal, nanometer‐scale Al coating on one surface, serving as an efficient infrared reflector without significantly altering film thickness or surface morphology.

### 4.4 Spectroscopic Analysis

The transmittance and reflectance of the samples across the IR optical properties within the 2.5–14 µm range were analyzed using a PerkinElmer Spectrum One FT‐IR spectrometer integrated with an upward‐facing IntegratIR sphere module (PIKE Technologies) for accurate diffuse and specular measurements.

### 4.5 Scanning Electron Microscope (SEM)

SEM analysis was carried out using Genesis‐2020 SEM.

### 4.6 Differential Scanning Calorimetry (DSC)

DSC analysis was conducted on the W‐VO_2_ powder using a TA Instruments Discovery DSC system. The thermal transition behavior was evaluated over a temperature range of 0°C–150°C at a heating rate of 10 °C/min, utilizing a liquid nitrogen cooling unit.

### 4.7 X‐Ray Diffraction Analysis (XRD)

XRD analysis was conducted using Multi‐Purpose High Performance X‐ray Diffractometer [PANalytical (X'PERT‐PRO Powder)].

### 4.8 Wettability

The water contact angle was measured using a 6 µL droplet of deionized water on a Phoenix‐10 goniometer (SEO Co., Ltd.).

### 4.9 Mechanical Stability

Stress‐strain behavior and flexibility of the AMF were evaluated using SALT/ST‐100 (Salt Co., Ltd.).

## Conflicts of Interest

The authors declare no conflicts of interest.

## Supporting information




**Supporting File**: smll74620‐sup‐0001‐SuppMat.docx.

## Data Availability

The data that support the findings of this study are available on request from the corresponding author. The data are not publicly available due to privacy or ethical restrictions.
