# Supplementary material for: Adaptive Metafilm With Scalable Phase‐Tunable Emissivity for Energy‐Efficient Thermal Management
Source: Small. 2026 Jul 25;22(50):e74620. doi: 10.1002/smll.74620 (PMC13548933; doi:10.1002/smll.74620)
Supplement: Supplementary file 1 [file SMLL-22-e74620-s001.docx]

**Adaptive Metafilm with Scalable Phase-Tunable Emissivity for Energy-Efficient Thermal Management**

James Laurence Ruello^1^, Sudip Kumar Pal^1^, Xiaoqing Yu^1^, Joonyoung Yoo^1^, Sihyun Jang^1,3^, Gayeong Kim^2^, Gunwoo Kim^1,2,3*^

1 Department of Organic Materials and Textile Engineering, Jeonbuk National University, 567 Baekje-daero, Deokjin-gu, Jeonju-si, Jeonbuk, 54896, Republic of Korea
2 Department of Carbon Materials and Fiber Engineering, Jeonbuk National University, 567 Baekje-daero, Deokjin-gu, Jeonju-si, Jeonbuk, 54896, Republic of Korea
3 Department of Carbon Composites Convergence Materials Engineering, Jeonbuk National University, 567 Baekje-daero, Deokjin-gu, Jeonju-si, Jeonbuk, 54896, Republic of Korea

*Corresponding author. E-mail address: creaventure@jbnu.ac.kr (G. Kim)

Tel.: +82 63 270 2356

**Figure S1.** Schematic diagram of the fabrication of the adaptable metafilm and its configuration.

**Figure S2.** Theoretical absorption profile of AMF at varying thickness. (a) 20 µm, (b) 80 µm, (c) 100 µm, (d) 120 µm, and (e) 150 µm.

**Figure S3.** Theoretical scattering efficiency profile of AMF at varying thickness. (a) 20 µm, (b) 50 µm (c) 80 µm, (d) 100 µm, (e) 120 µm, and (f) 150 µm.

**Figure S4.** SEM images of the (a) Al-sputtered surface of the AMF and its (b) cross-section.

**Figure S5.** Transmittance comparison between high and low temperatures using adaptive with **0.5%W-VO_2_:1% ITO** concentration at varying thickness. (a) 30 µm, (b) 40 µm, (c) 50 µm, (d) 60 µm, (e) 70 µm, (f) 80 µm, (g) 90 µm, (h) 100 µm, and (i) 110 µm.

**Figure S6.** Transmittance comparison between high and low temperatures using adaptive with **1% W-VO_2_:1% ITO** concentration at varying thickness. (a) 40 µm, (b) 50 µm, (c) 60 µm, (d) 70 µm, (e) 80 µm, (f) 90 µm, (g) 100 µm, and (h) 110 µm.

**Figure S7.** Transmittance comparison between high and low temperatures using adaptive with **2% W-VO_2_:1% ITO** concentration at varying thickness. (a) 40 µm, (b) 50 µm, (c) 60 µm, (d) 70 µm, (e) 80 µm, (f) 90 µm, (g) 100 µm, (h) 120 µm, and (i) 130 µm.

**Figure S8.** Transmittance comparison between high and low temperatures using adaptive with **5% W-VO_2_:1% ITO** concentration at varying thickness. (a) 30 µm, (b) 50 µm, (c) 60 µm, (d) 70 µm, (e) 80 µm, (f) 90 µm, (g) 100 µm, (h) 110 µm, and (i) 120 µm.

**Figure S9.** Transmittance comparison between high and low temperatures using adaptive with **7% W-VO_2_:1% ITO** concentration at varying thickness. (a) 40 µm, (b) 50 µm, (c) 60 µm, (d) 70 µm, (e) 80 µm, (f) 90 µm, (g) 100 µm, and (h) 110 µm.

**Figure S10.** Transmittance comparison between high and low temperatures using adaptive with **5% W-VO_2_:0.5% ITO** concentration at varying thickness. (a) 40 µm, (b) 50 µm, (c) 60 µm, (d) 70 µm, (e) 80 µm, (f) 90 µm, and (g) 100 µm.

**Figure S11.** Transmittance comparison between high and low temperatures using adaptive with **5% W-VO_2_:2% ITO** concentration at varying thickness. (a) 30 µm, (b) 40 µm, (c) 50 µm, (d) 60 µm, (e) 70 µm, (f) 80 µm, (g) 90 µm, (h) 100 µm, and (i) 110 µm.

**Figure S12.** Transmittance tunability of AMF at varying thickness using different W-VO_2_: ITO concentrations. (a) 0.5%W-VO_2_:1% ITO, (b) 1%W- VO_2_:1% ITO, (c) 2%W- VO_2_:1% ITO, (d) 7%W- VO_2_:1% ITO, (e) 5%W- VO_2_:0.5% ITO, and (f) 5%W- VO_2_:2% ITO.

**Figure S13.** IR spectral reflectance of 50 µm thick aluminum-sputtered PE at low and high temperatures.

**Figure S14.** (a) Durability test showing IR reflective performance of AMF after 100 bending cycles. (b) UV aging test of AMF at high and low temperatures.

**Figure S15.** Thermal images of PDMS and AMF on metal and bare metal samples at increasing temperature exposures.
